# Advances in the biochemical diagnostics of primary aldosteronism: from immunoassays to steroidomics and proteomics

**DOI:** 10.3389/fendo.2025.1548344

**Published:** 2025-04-16

**Authors:** Alicja Szatko, Agata Toboła, Henrik Falhammar, Wojciech Zgliczyński, Piotr Glinicki

**Affiliations:** ^1^ EndoLab Laboratory, Centre of Postgraduate Medical Education, Warsaw, Poland; ^2^ Department of Endocrinology, Centre of Postgraduate Medical Education, Warsaw, Poland; ^3^ Doctoral School of Translational Medicine, Centre of Postgraduate Medical Education, Warsaw, Poland; ^4^ Department of Endocrinology, Karolinska University Hospital, Stockholm, Sweden; ^5^ Department of Molecular Medicine and Surgery, Karolinska Institutet, Stockholm, Sweden

**Keywords:** primary aldosteronism, hypertension, adrenal tumor, aldosterone, steroidomics, proteomics, immunoassays, biomarkers

## Abstract

Primary aldosteronism is the most common cause of secondary hypertension, yet most cases remain unrecognized and left without optimal treatment. The diagnostic inertia may be attributed to the lack of specific symptoms, insufficient awareness among physicians, still conflicting indications for screening for primary aldosteronism and first and foremost challenging diagnostics. This review describes the current challenges of biochemical diagnostics of primary aldosteronism, including screening, case confirmation and subtyping. It also discusses immunoassays widely used in assessment of suspected autonomous aldosterone secretion – recent advances in the field and limitations of the method in comparison to the gold standard - liquid chromatography –tandem mass spectrometry. The review focuses on the application of novel “omics” strategies in the diagnostics of primary aldosteronism. Steroidomics and proteomics offer a possibility to simultaneously assess steroids and protein/peptides on a large scale. This multianalyte approach in comparison to the selective quantification of a chosen compound has been proved useful in the diagnostics of primary aldosteronism. It also offers a unique insight into the individual characteristics, underlying mechanisms and even reflects the genetic alterations of primary aldosteronism cases. The “omics” techniques are associated with large amounts of generated data, the interpretation of which may be troublesome and often necessitates the use of artificial intelligence. The novel advances in the biochemical diagnostics of primary aldosteronism, including “omics” techniques, presented in this review may help to address the most emerging problems, increase the number of diagnosed patients and facilitate the choice of an optimal treatment.

## 1 Introduction

Primary aldosteronism (PA) is caused by autonomous aldosterone secretion from the zona glomerulosa of adrenal cortex (1). Aldosterone excess leads to increased sodium and water resorption in the distal tubule and collecting duct of the nephron (1, 2). Water and sodium overload suppresses renin release from the renal juxtaglomerular cells (1). Thus, increased aldosterone-to-renin ratio, together with hypertension and hypokalemia, is the hallmark of PA. The aldosterone-to-renin ratio is widely used in screening for PA.

PA is the most common curable form of secondary hypertension, yet it remains widely unrecognized, with fewer than 2% of patients at-risk ever tested and half of those patients diagnosed and treated (1, 3, 4). The reasons why PA is widely overlooked include lack of awareness among clinicians, absence of characteristic symptoms and often burdensome multi-step diagnostics and subtyping process (1). Patients at younger age, with high systolic and diastolic blood pressure (BP), and hypokalemia are more likely to be screened for PA than older patients with relevant comorbidities (5).

The problem of PA underdetection is even more prominent when taken into consideration that the detrimental effects of aldosterone excess are partially independent from the influence of the hypertension caused by PA (1, 6, 7). Patients with PA are more prone to develop left ventricle hypertrophy, increased aortic stiffness, dysfunction of endothelium, albuminuria and hyperfiltration, when compared to BP-matched controls (7–10). PA is also associated with higher prevalence of metabolic syndrome and type 2 diabetes mellitus (7). Furthermore, aldosterone excess contributes to increased urinary calcium loss and hypocalcemia, which translates into higher prevalence of bone fractures and osteoporosis in patients with PA (11, 12).

The established diagnostic workup for PA is a costly, complex process that usually requires multiple visits before the diagnosis is finally confirmed. Thus, mainly patients with high probability of PA and unequivocal diagnostic results undergo screening. Further diagnostics (including confirmatory testing and adrenal venous sampling (AVS) to differentiate between uni- and bilateral subtype) is often burdensome and usually limited to large tertiary centres. As a result, the majority of patients with PA never receive the correct diagnosis (13). Therefore, novel tools and techniques are needed to improve diagnostic stratification and subtyping for PA.

This review provides an overview of recent advances in biochemical diagnostics of PA, including cutting-edge methods of steroidomics and proteomics, with a focus on targeted approaches. These techniques provide “a snapshot” of large numbers of released steroids, metabolites and proteins/peptides at the given time. The global analysis of compound group enables a comprehensive understanding of the underlying mechanisms of PA, but also unveils novel diagnostic options.

## 2 Diagnostics of primary aldosteronism: current approaches and challenges

According to the Endocrine Society Guidelines, indications of PA screening include patients with: (1) BP above 150/100 mmHg on repeated (at least three) measurements, (2) resistant hypertension, (3) hypertension controlled on 4 or more hypertensive agents, (4) hypertension with hypokalemia (both spontaneous and diuretic-induced), (5) hypertension and adrenal mass, (6) hypertension and obstructive sleep apnea (OSA), (7) hypertension and family history of cerebral vascular event (CVA) or hypertension at the age younger than 40 years, and (8) all patients with hypertension and a first-degree relative diagnosed with PA (14). The authors of a position statement and consensus of the Working Group on Endocrine Hypertension of the European Society of Hypertension recommend that the screening for PA should also include patients with hypertension and atrial fibrillation (AF) not associated with structural heart disease, since the prevalence of AF among patients with PA is nearly four times higher than among subjects with resistant hypertension (15, 16). However, the indication for PA screening in patients with hypertension and OSA has been questioned based on the results of the multiethnic, cross-sectional HYPNOS study (Hyperglycemic Profiles in Obstructive Sleep Apnea) (15, 17).

Recommendations published by the national endocrinology societies also apply non-uniform criteria for PA screening. French Endocrinology Society (SFE), French Hypertension Society (SFHTA) and Francophone Endocrine Surgery Association (AFCE) highlight the need for PA screening in patients with hypertension and disproportionate target organ damage (18). The recently published British and Irish Hypertension Society (BIHS) statement on diagnosis and management of PA recommends facilitated diagnostic strategy for screening for PA (19). According to BIHS, clinical situations in which PA screening should be introduced include resistant hypertension, hypertension with hypokalemia, hypertension with adrenal mass, and hypertension in adults below 40 years (19). However, there are some investigators advocating for screening for PA of all hypertensive patients (20). The rationale behind this approach includes considerable health benefits for diagnosed patients with PA after tailored treatment and the possibility to avoid the confounding effect of antihypertensive medications on aldosterone and renin measurements (20).

Alongside the lack of uniform indications for PA screening, recommended aldosterone-to-renin ratio (ARR) as a screening test is subject to several limitations (14). Renin may be assessed as plasma renin activity (PRA) evaluating renin enzymatic activity to generate angiotensin I from angiotensinogen under controlled conditions over time, or direct renin concentration (DRC) based on the measurement of renin and active prorenin concentration in plasma. Measurement of renin may be challenging, mainly due to low concentration and instability in refrigerated temperature, which leads to cryoactivation of prorenin to renin and falsely elevated results (21, 22). Thus, it is recommended to transport the probes (usually EDTA plasma sample collection tubes are used) at room temperature to the laboratory up to 30 min after blood collection, centrifugate before the immediate automated DRC determination (CLIA, Chemiluminescent immunoassays) or centrifugate, quickly freeze and store frozen plasma prior to the postponed manual DRC measurement (IRMA, Immunoradiometric assay and ELISA, enzyme-linked immunosorbent assay) (14). For the PRA determination, routinely EDTA plasma sample collection tubes are used, samples should be transported on ice to the laboratory within 30 min from the blood collection, centrifugated (4°C), frozen and stored till the manual PRA measurement (commonly radioimmunoassay (RIA) or enzyme immunoassay (EIA, ELISA) methods are used). Immunoassays are widely used for determination of DRC and PRA. Despite convenience and short turnaround time, DRC and PRA immunoassays may lack sensitivity and exhibit low antibody storage stability and cross-reactivity with multiple structurally similar angiotensin-like peptides (21, 23). However, angiotensin I for PRA calculation may also be measured by liquid chromatography–tandem mass spectrometry (LC-MS/MS), which offers high accuracy of angiotensin I measurement at low concentrations (often observed in PA) and allows for determination of metabolites (e.g., angiotensin II, angiotensin III, angiotensin IV) for the broader assessment of the renin‐angiotensin‐aldosterone system (RAAS) (21, 23). Aldosterone can also be measured in serum or plasma. For the serum aldosterone concentration determination clot activator serum sample collection tubes are routinely used, while EDTA plasma sample collection tubes are used when plasma is analysed. Contrary to DRC, the probes for aldosterone determination are not sensitive to temperature drop. Moreover, differences depend on the type of biological material (serum/plasma) used to determine blood aldosterone levels. Aldosterone concentration can be determined by various immunoassays or LC-MS/MS technique. However, the results obtained using LC-MS/MS are 30% lower when compared to radioimmunoassay aldosterone measurement (24, 25). Since the results of aldosterone determination differ significantly depending on the used method (RIA, EIA/ELISA, chemiluminescence immunoassay (CLIA, ECLIA), LC-MS/MS technique), assay-specific thresholds should be used when ARR (or ADRR (aldosterone-to-direct-renin ratio) if DRC is used) is calculated (25, 26).

Since the advantage of ARR in the screening for PA was shown by Hiramatsu et al., ARR screening has been used widely (27). Most commonly used cutoff values are 30 for ARR (ng/dL/ng/mL/h) and 3.7 for ADRR (ng/dL/mU/L) (14). Other proposed screening tests for PA, e.g., the aldosterone-to-angiotensin II ratio using commercial ELISA set showed worse diagnostic performance (28). However, the interpretation of ARR should include various factors and limitations. Firstly, as presented in the meta-analysis by Hung et al., assessing ARR diagnostic performance, no single ARR threshold for PA screening could be recommended (29). Furthermore, nearly all commonly used antihypertensive drugs interfere with RAAS, leading to false positive or false negative results of ARR (14). Not only do antihypertensive agents influence ARR, but also non-steroidal anti-inflammatory drugs (NSAIDs), estrogen-containing contraceptives, hormone replacement therapy (HRT) and selective serotonin reuptake inhibitors (SSRIs) may alter the result (14, 30). False positive results of ARR may also be observed in older age, impaired kidney function, increased dietary salt intake, luteal phase of menstrual cycle and in patients with the extremely rare disorder Gordon syndrome (or pseudohypoaldosteronism type 2) (31). False negative results may be a consequence of pregnancy, hypokalemia, salt intake restriction, vomiting, diarrhoea, malignant or renovascular hypertension (31). In other conditions such as renal pseudohypoaldosteronism type 1 the ARR can be high with very high aldosterone concentrations, but renin concentrations are not suppressed, and the patients do not have hypertension (32).

Bloods for aldosterone and renin measurement should be drawn in the morning, after 2hr in upright position, then permitted to settle (sit for 5 to 15 minutes) before the blood collection (14, 33). Suboptimal screening conditions may, together with other pre-analytical errors (e.g., during specimen collection, handling and transportation to the laboratory), markedly influence the results. Despite measurement in optimal conditions, ARR shows high within-patient variability (34). Thus, in patients with high pretest probability of PA and not elevated ARR, it should be repeated at least twice (34, 35). Suppressed renin concentration often leads to false positives, even if the aldosterone concentration is low. Hence, it has been suggested to use a minimum aldosterone concentration of >15 ng/dL (>415 pmol/L) to be able to interpret a positive test (14). However, it may lead to underdiagnosing some individuals with PA, especially patients with PA and bilateral adrenal hyperplasia (BAH) (14, 21). Thus, some investigators suggest a cut-off of 5 ng/dL (138.7 pmol/L) for aldosterone concentration, especially when assessed by LC-MS/MS (36).

Once a positive ARR is confirmed, lack of aldosterone suppression should be demonstrated in one of four tests (saline infusion test, oral sodium loading test, fludrocortisone suppression test or captopril challenge test) (14). All these tests can be done in an outpatient setting, also fludrocortisone suppression test, even though the latter in most centres is done as an inpatient test (37). In patients with suppressed renin concentration, spontaneous hypokalemia, and plasma/serum aldosterone concentration ≥ 20 ng/dL (≥ 555 pmol/L) confirmatory test is not needed (14). Similarly to the previous stages of PA diagnostics, there is a remarkable heterogeneity in confirmatory testing protocols and interpretation of the results (38). Notably, the use of confirmatory tests is not evidence-based, since none of the studies advocating their use in confirmation of PA diagnosis has met state-of-the-art criteria used for validation of diagnostic tests (19, 39–42).

After the diagnosis of PA, optimal treatment (unilateral adrenalectomy or pharmacotherapy with mineralocorticoid receptor antagonist) should be initiated, depending on the source (unilateral/bilateral) of autonomous aldosterone secretion, surgical candidacy, and patient preference (14, 43). Unenhanced adrenal computed tomography (CT) may assist in treatment planning, although it is associated with substantial number of false positive (identifying non-functional adrenal adenomas incorrectly linked to autonomous aldosterone secretion) or false negative results (underdetection of small lesions), except in patients below 35 years old, with spontaneous hypokalemia, aldosterone concentration >30 ng/dL (>831 pmol/L), and unilateral adrenal lesion measuring ≥ 10 mm, in whom diagnostic accuracy of CT is high and AVS is not necessary (14, 43). Overall, the concordance rate of CT and AVS in patients with unilateral disease was 50% (44). Thus, the majority of patients need to undergo AVS for subtyping. Although AVS is characterized by high sensitivity (95%) and specificity (100%) in detecting unilateral aldosterone hypersecretion, it is a costly, invasive, highly specialized procedure that should be performed only in experienced centres (14, 45). It should be noted that canulating the right adrenal vein is very difficult with low rate of success in non-experienced hands.

**Figure 1:**
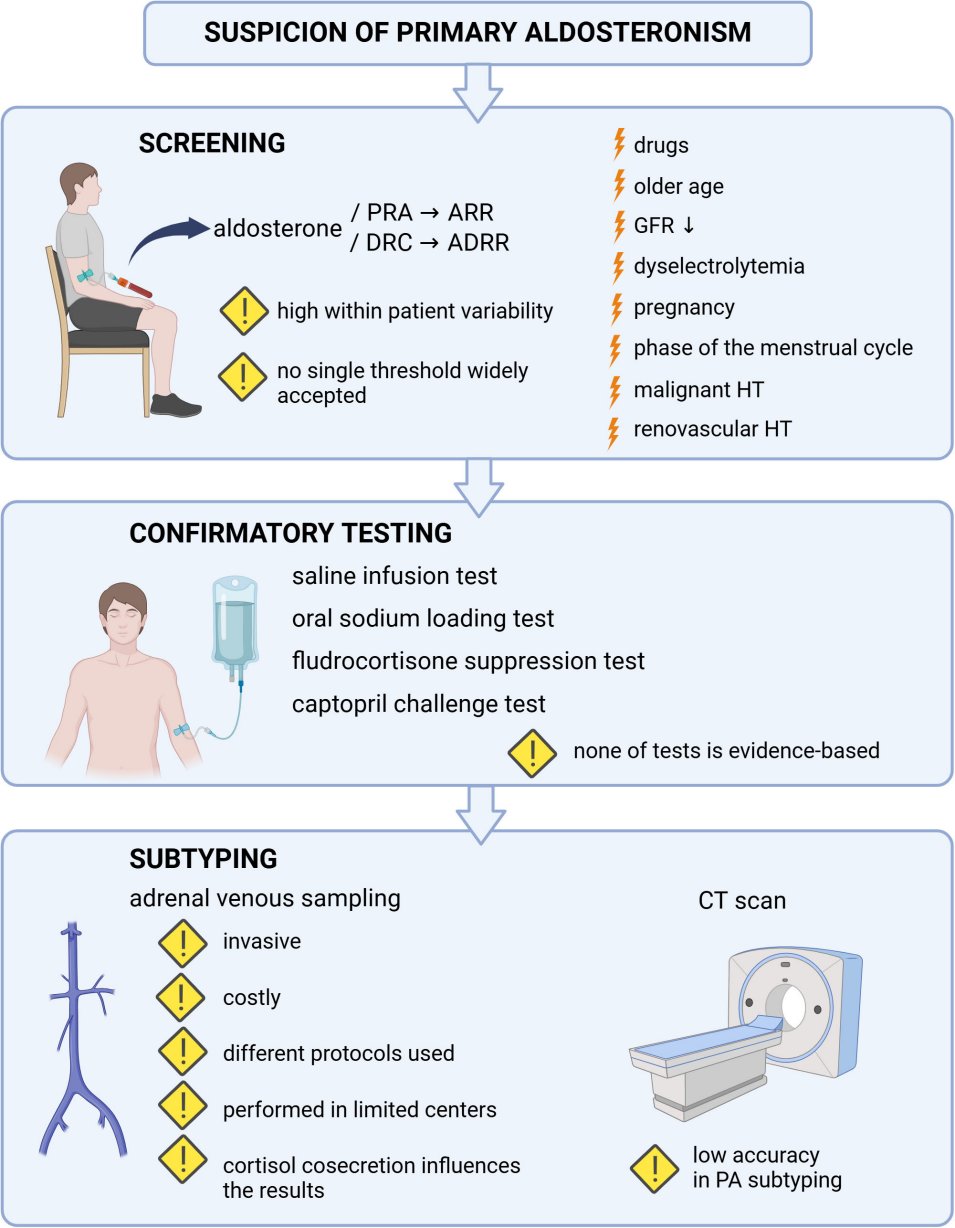
The limitations of routine diagnostics of primary aldosteronism. Factors which may influence the result of primary aldosteronism screening were marked with the lightning sign. Exclamation mark was used to underline the limitations of screening, confirmatory tests and subtyping of primary aldosteronism. PRA, plasma renin activity; ARR, aldosterone-to-renin ratio; DRC, direct renin concentration; ADRR, aldosterone-to-direct-renin ratio; GFR, glomerular filtration rate; HT, hypertension; CT, computed tomography; PA, primary aldosteronism. Created in BioRender. BioRender.com/n46h901

Thus, all the listed limitations of routine diagnostics of PA (**Figure 1**) indicate the necessity to establish novel, improved diagnostic strategies.

## 3 From immunoassays to mass-spectrometry

For many years, immunoassays, in particular RIA and CLIA, have been the primary method used for measuring selected steroids in plasma/serum and urine. Nonetheless, immunoassay, which is based on the reaction of an antigen with a specific antibody, has multiple limitations. These include limited sensitivity and reproducibility, especially at lower concentrations. Currently, mass-spectrometry based techniques (including gas chromatography – mass spectrometry (GC/MS) and LC–MS/MS) are considered the gold standard for quantification of steroids in biological fluids (46, 47). LC–MS/MS combines liquid chromatography separation of the particles followed by their mass-based detection. Its high sensitivity and specificity result from minimizing interference from cross-reactivity and non-specific reactions. Its relevant diagnostic value in PA has been reported in a meta-analysis (46).

Furthermore, the LC-MS/MS technique allows simultaneous quantification of multiple compounds and is a time-efficient analysis. Noh et al. demonstrated successful measurements of adrenocortical steroids, catecholamines and plasma free metanephrines in a single run (48). Derivatization with alkyl chloroformates allowed the protection of polar and hydrophilic groups of medullary amines and resulted in a satisfactory signal-to-noise ratio and peak shape, while maintaining effective steroid quantifications (48).

One of the limitations of the LC-MS/MS technique includes laborious sample preparation. Over the past few years, this issue has been addressed in multiple studies and increasingly faster measurement methods using LC-MS/MS have been developed. By improving the efficiency of acid hydrolysis, Yin et al. developed an assay of rapid aldosterone measurement in samples from 24hr urine collections (49). Ultra-performance liquid chromatography-tandem mass spectrometry (UPLC-MS/MS) has also been tested in aldosterone quantification in plasma – this method enables rapid aldosterone measurement, while maintaining high accuracy (50).

Nevertheless, given the limited availability of LC-MS/MS in many centres and countries, there has arisen a need to improve existing, widely accessible methods to ensure better diagnostics and the ability to compare research results with those obtained using the LC-MS/MS technique. A new, two-site sandwich chemiluminescent enzyme immunoassay (CLEIA) to automatically measure both plasma/serum aldosterone concentration and active renin concentration (ARC), using monoclonal antibodies immobilized onto ferrite particles, has been implemented into clinical practice (51, 52). Nishikawa et al. compared LC-MS/MS with CLEIA and conventional RIA immunoassays for aldosterone measurement in the blood (53). The median aldosterone concentration of the LC-MS/MS corresponding to RIA established PA criterion was almost 2.5 times lower for LC-MS/MS technique, while CLEIA values were similar to LC-MS/MS. A study conducted by Kobayashi et al. (54) showed that the plasma aldosterone concentration measured by CLEIA was significantly lower than the value obtained with RIA, and new cut-offs for screening and confirmatory tests using CLEIA for the diagnosis of PA were suggested. Thus, the good linearity over a wide range of concentrations and accuracy comparable to LC-MS/MS may establish CLEIA as an alternative testing method in centres and countries without access to LC-MS/MS (51).

Despite the undeniable advantages of LC-MS/MS technique, its application is limited to selected centres and thus the overwhelming majority of routine diagnostics of PA still relies on the use of immunoassays. In the future, LC-MS/MS will not likely replace immunoassays in the PA diagnostics in many centres, thus ways to optimize the simultaneous use of both, diagnostic cut-offs, comparability and reproducibility will be necessary.

## 4 Targeted steroidomics

The rapidly evolving field of “omics” techniques allows for characterization of an entire set of chosen compounds produced by cell, tissue, organ or organism at the same time. Thus, profound assessment of multiple disorders at a resolution that has never been possible is now available. One of them, targeted steroidomics (the terminology was introduced by Sjovall in 2004), involves mass-spectrometric assessment of predefined steroids on a multianalyte approach, measured in blood (serum/plasma) or urine (24-hour urine collection) (55–57). Although the use of steroid profiling in the diagnostics of adrenocortical diseases has a history of more than half a century, only a recent development of liquid chromatography or gas chromatography coupled with mass spectrometry has transformed the investigation of adrenocortical conditions, unveiling unknown and underappreciated steroid players (56–60).

The application of steroidomics has proven useful in evaluation of adrenocortical cancer (ACC) and autonomous cortisol secretion (CS), but also PA (61–75). In the study of Berke et al., investigating the profile of 19 plasma steroids in 577 patients with adrenal incidentaloma, patients with PA were distinguished by high plasma concentrations of 18-oxocortisol, 18-hydroxycortisol, 18-hydroxycorticosterone and aldosterone (64). The utility of 18-hydroxycorticosterone in plasma and urinary “hybrid steroids” (combining the structural characteristics of aldosterone and cortisol): 18-hydroxycortisol and 18-oxocortisol were demonstrated to differentiate patients with PA from those with primary hypertension and normotensive subjects (65). A threshold for 24hr urinary 18-hydroxycortisol excretion greater than 330 μg/d together with positive ARR confirmed the diagnosis of PA without the need for further confirmatory/exclusion tests (65). A 24hr urinary 18-hydroxycortisol excretion greater than 510 μg/d distinguished the patients with aldosterone producing adenoma (APA), which together with identification of unilateral adrenal tumour on a CT scan may allow to proceed directly to unilateral adrenalectomy without AVS (65). Peripheral plasma 18-oxocortisol concentration measured by LC-MS/MS was also proved useful in discrimination of patients with APA from BHA (a cutoff value of 4.7 ng/dL was used with a sensitivity of 83% and specificity of 99%) (66).

**Figure 2:**
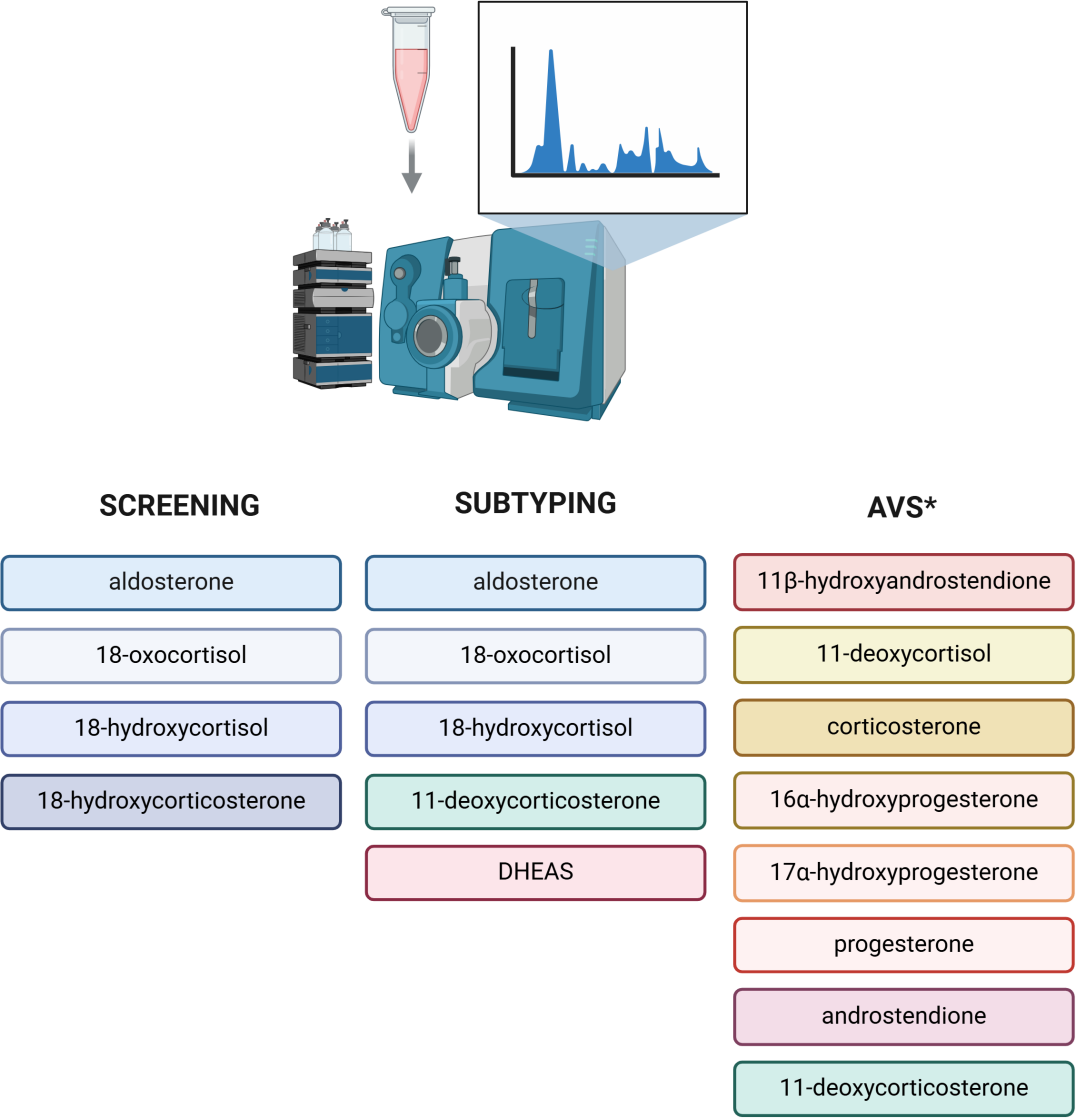
Plasma steroidomics in diagnostics of primary aldosteronism. Steroids demonstrating the highest discriminatory power in primary aldosteronism screening and subtyping. *Panel of 8 steroids characterized by higher selectivity index than cortisol both at baseline and after adrenocorticotropic hormone, which may improve the proportion of successful adrenal venous sampling [70]. DHEAS, dehydroepiandrosterone sulfate; AVS, adrenal venous sampling. Created in BioRender. BioRender.com/u15i195

The results of one study by Eisenhofer et al. demonstrated that the use of a 8 steroid panel in plasma (aldosterone, 18-hydroxycortisol, 18-oxocortisol, 11-deoxycoticosterone, cortisol, cortisone, dehydroepiandrosterone, and androstenedione) together with ARR was more effective than ARR alone for discriminating patients with PA from those with primary hypertension (67). Among assessed steroids, aldosterone, 18-oxocortisol, and 18-hydroxycortisol were characterized by the highest discriminatory power (67). This approach combined with machine learning also allowed to differentiate the patients with APA associated with *KCNJ5* variants with the sensitivity of 85% and the specificity of 97% (67). In another study published by Eisenhofer et al. on 216 patients with PA, concentrations of 18-oxocortisol in plasma was 8.5 times higher in patients with APA, than in those with BHA (68). However, the area under the curve (AUC) of 0.659 showed limited utility of that steroid in subtyping PA (68). The accuracy was remarkably improved to AUC 0.889 by analysing the whole panel of 15 steroids, emphasizing the potential utility of mass-spectrometry-based measurement of multiple steroids rather than analysing them individually (68). The diagnostic application of the steroid profile of peripheral venous plasma measured by LC-MS/MS was also the subject of the study by Yang et al. (69). The investigators targeted distinguishing the patients with BHA from those with macro-APA (diameter of adrenal lesion ≥ 10 mm) and micro-APA (diameter < 10 mm), the latter often undetectable on CT scans (69). The obtained results revealed distinct differences in steroid profiles between compared groups, with aldosterone, 18-oxocortisol, 18-hydroxycortisol and dehydroepiandrosterone sulphate (DHEAS) having the highest differentiating value (69). Interestingly, the diagnostic performance of steroid probability score for PA (based on mass-spectrometric assessment of steroids in plasma integrated by machine-learning tools) was not influenced significantly by the interfering antihypertensive drugs (AUC 0.848 with the use of antihypertensive medications; AUC 0.893 without antihypertensive medications with the proven impact on the RAAS), as it was observed for ARR (AUC 0.765 with and AUC 0.845 without the interfering with RAAS antihypertensive drugs) (70). Plasma steroids which showed the highest discriminatory power in primary aldosteronism screening and subtyping are presented in **Figure 2**.

Apart from the assessment of plasma steroidomics, quantification of steroid metabolites excretion in 24hr urine collection was proven useful in identification and subtyping of PA (71). The choice of urine as a sample matrix has advantages over plasma/serum: non-invasive sample collection, not requiring any medical professionals, thus lower cost, unaffected by alterations in supine vs. seated blood sampling, and in case of 24hr urine collection: comprehensive measurement of steroid output, independent from diurnal steroid differences (56, 67, 71). However, 24hr urine collection can be inconvenient and difficult for some patients, and in those blood samples could be preferred. There are also substantial differences in sample preparation, depending on the matrix used: serum/plasma or urine. Contrary to blood (plasma), in urine steroids are present as conjugates (glucuronides and sulphates). Thus, after extraction and prior to e.g., derivatization and analysis using GC/MS, conjugated steroids need to be hydrolysed (71). To remove those charged moieties different strategies are applied resulting in significant alterations in desired metabolites concentrations varying between the studies (72).

In the study of Prete et al., the assessment of 34 steroids (including mineralocorticoid, glucocorticoid, androgen, hybrid steroid metabolites and their precursors) in 24hr urine sample measured by GC/MS and analysed by machine-learning methods had an excellent accuracy (AUC 0.970) for the distinguishing of patients with PA from controls with normotension (73). Among assessed metabolites, 3α, 5β-tetrahydroaldosterone, tetrahydro-11-deoxycortisol, and 18-hydroxy-tetrahydro-11-dehydrocorticosterone had the highest discriminative value (73). Interestingly, their 24hr urinary output interquartile ranges did not overlap between patients with PA and controls (73). Conversely, computational analysis of urine steroidomics showed suboptimal diagnostic performance (AUC 0.650) in distinguishing patients with APA from those with BHA, probably due to high heterogeneity of the groups (73). However, analysis of 34 steroids integrated by machine learning approach with generalised matrix relevance learning vector quantization, allowed to distinguish patients with APA harbouring *KCNJ5* variants from other patients with PA with a high diagnostic accuracy (AUC 0.830) (73).

Multisteroid approach was tested as a tool not only to identify and subtype PA based on the analysis of serum/plasma or urine, but also to improve the diagnostic performance of AVS. Cortisol, used in interpretation of AVS results, has several disadvantages including longer half-life than aldosterone and fluctuations of cortisol concentration during AVS (74). Additionally, mild autonomous cortisol secretion (MACS) is often found in patients with PA which may influence AVS result (74, 75). In a study by Turcu et al., among 17 measured steroids, 8 steroids showed significantly higher selectivity index (SI, calculated as adrenal vein/inferior vena cava steroid concentration) than cortisol, both at baseline and after adrenocorticotropic hormone (ACTH) simulation (**Figure 2**) (74). Importantly, the use of 11ß-hydroxyandrostendione, corticosterone and 11-deoxycortisol allowed to rescue the majority of unsuccessful baseline catheterizations (with SI <2 for cortisol) (74). In a study by Chang et al., the application of steroid profiling measured by LC-MS/MS rescued 45% unstimulated and 66% ACTH stimulated unsuccessful cases of AVS based on immunoassay assessment (76). Furthermore, steroid profiling using LC-MS/MS allowed to identify 31% more cases of unilateral PA in comparison to widely used immunoassay (76). Thus, the application of steroid profiling may significantly improve the number of successful AVS and diagnostic accuracy of that procedure (77). Nevertheless, the obtained results should be interpreted cautiously, while the application of LC/MS-MS is only being tested in context of AVS, in which the concentrations of steroids are significantly higher than those measured in the blood.

Overall, mass-spectrometric assessment of steroid profiling has improved the understanding of the role of steroids in PA, going far beyond aldosterone. The recently published studies have revisited the importance of “hybrid steroids” as PA biomarkers. The use of targeted steroidomics integrated by machine learning tools may streamline the identification and subtyping of patients with PA. The application of novel methods such as high-resolution matrix-assisted laser desorption/ionization mass-spectrometry (MALDI-MS) was proven useful to integrate metabolomic data with spatial information obtained from standard histology, resulted in better understanding of functional anatomy of APA, defining genotype-phenotype correlations and discovering new biomarkers (78). Furthermore, the application of untargeted metabolomics may open a new chapter in understanding the pathophysiology of PA unveiling alterations in metabolic pathways and defining novel diagnostic biomarkers (79).

## 5 Targeted proteins and peptides analysis methods, untargeted proteomics

Simultaneously with the quantification of multiple steroids using mass spectrometry in the diagnostics of PA, extensive research has been conducted in the field of proteomics. Targeted proteomics allows for precise quantification of preselected proteins and peptides, while untargeted proteomics aims to quantify all detectable proteins and is associated with laborious work of identifying them. Proteomics examines the structure and function of peptides and proteins involved in the physiological and pathophysiological processes of RAAS, which could be an alternative to PRA/DRC. Recent studies evaluated RAAS equilibrium using LC-MS/MS to quantify the angiotensin peptidome (including angiotensin I, angiotensin II, angiotensin III, angiotensin IV, angiotensin (1–7), and angiotensin (1–5)) simultaneously from a single sample (80–82). The main principle of RAAS equilibrium analysis is based on the incubation of prestored frozen serum at the temperature of 37°C, controlled pH 7.4 for 1 hour without addition of any substances interfering with angiotensin production or degradation, which leads to establishment of equilibrated status of RAAS (82). Thus, the equilibrium angiotensin II (eqAngII) concentration is the resultant of the activity of all angiotensin processing enzymes in the probe, once the equilibrium is established (82). The assessment of eqAngII and calculation of aldosterone-to-angiotensin II ratio may be a promising tool in PA diagnostics, allowing to bypass often cumbersome PRA or DRC determination (82).

Prorenin, a precursor of renin, has been suggested to be valuable in the diagnostics of PA. Compared with active renin, prorenin is characterized by stable release, not affected by multiple stimuli including change of body position (83). The (pro)renin receptor ((P)RR) levels were found to be positively correlated with aldosterone synthase (CYP11B2) concentrations in APA tissues, plasma aldosterone concentrations and urinary aldosterone excretion, suggesting its role in aldosterone synthesis (84). Nevertheless, in the same study, serum (P)RR was neither associated with plasma aldosterone concentration nor adrenal (P)RR expression level (84).

Beyond the direct assessment of RAAS components, other peptides and proteins, primarily associated with, e.g., inflammation or transmembrane transport, were investigated as potential biomarkers of PA. Proteins and peptides from the granin family involved in the regulated secretory pathway (packaging, storage, and release of peptide hormones and neurotransmitters) are currently being studied as a potential biomarkers of PA. The results of the study by Glinicki et al. on 10 patients with PA and 22 patients with non-functional adrenal adenoma (NFAA) demonstrated that among 10 proteins and peptides from the granin family, pancreastatin (pancreastatin/chromogranin A (250-301aa) -amide peptide) and secretoneurin (a small 33aa peptide of secretogranin II (SgII)/chromogranin C (CgC) (1-617aa)) were differentiating those two groups (85). Recently, extracellular vesicles (EVs), biological nanostructures released from all cells, have also become a compelling subject of interest for researchers worldwide (86). Given that EVs transport various biomolecules including multiple proteins, lipids, nucleic acids derived from parent cells, they hold the potential to serve as a source of biomarkers for various diseases, including PA (87).

Recent studies investigated the role in PA diagnosis of serum and urinary alpha-1-acid-glycoprotein (AGP1 or A1G1), also known as orosomucoid protein 1 (ORM1), an acute-phase protein associated with inflammation (87–90). The notable upregulation of AGP1 in urinary extracellular vesicles (uEVs) in PA was studied by Barros et al., who investigated the proteome of patients with PA, searching for mediators associated with renal and extrarenal damage induced by chronic elevated aldosterone concentration (89). Sequential ultracentrifugation was applied for isolation of uEVs, then the International Society for Extracellular Vesicles guidelines were used to describe isolated EVs, using transmission electron microscopy (TEM), immunoblotting, and nanoparticle tracking analysis (NTA) (89, 91). A considerable upregulation of AGP1 in patients with PA (2.43-fold increase) was observed in the comparison to the control group, however, the limited size of a study (7 patients with PA and 8 healthy controls) necessitates interpreting the results with caution (89). Elevated concentration of serum AGP1 in patients with PA compared with individuals with essential hypertension and normotensive controls was also demonstrated by Carvajal et al. (90). Interestingly, the concentrations of other inflammatory markers: high sensitive C-reactive protein (hs-CRP), plasminogen inhibitor activator-1 (PAI-1), matrix metallopeptidase 9 (MMP-9) and malondialdehyde (MDA), free cystatin-C (CysC), neutrophil gelatinase associated lipocalin (NGAL or LCN2), and interleukin 6 (IL-6) did not differ between the groups (90).

Regarding the influence of aldosterone on NaCl-transporting proteins in renal tubules, Ochiai-Homma et al. analysed the quantitative changes in pendrin, a Cl^−^/HCO_3_^–^ exchanger protein, in uEVs isolated from patients with PA and from a rat model of aldosterone excess (92). Pendrin was found in uEVs in humans and rats (92). In a rodent model, its levels, as well as epithelial Na^+^ channel (ENaC) and Na^+^Cl^–^cotransporter or thiazide-sensitive sodium chloride cotransporter (NCC) levels in uEVs, were correlated with renal abundance (92). Interestingly, pendrin levels in uEVs were reduced by 49% after adrenalectomy or pharmacological mineral receptor blockade (92). The role of abovementioned uEVs NCC in PA was also studied by Kong et al., who aimed to identify biomarkers helping to distinguish PA subtypes without AVS procedure (92, 93). The promising use of phosphorylated form of NCC (pNCC) in non-invasive PA subtyping was noted (93). In this study, spot urine samples from 50 patients with PA who underwent AVS were compared within the low lateralization index (l-LI) group and high lateralization (h-LI) index group (93). NCC and pNCC were more abundant in the h-LI group (93). Furthermore, somatic *KCNJ5* variants were detected in 65.4% of the APA cases, and carriers of somatic *KCNJ5* variants compared with non-carriers had a higher abundance of pNCC in uEVs (93). Positive correlation between pNCC abundance and plasma aldosterone concentration was demonstrated (93). However, the results should be confirmed in larger studies and evaluation of NCC as well as pNCC concentrations before and after treatment of PA would also be beneficial (93). The correlation between PAC and NCC adjusted by CD9 protein level in uEVs was found by Hayakawa et al. as well (94). Nevertheless, γ-epithelial sodium channel (ENaC) adjusted by CD9 protein level in uEVs was found to better correlate with plasma aldosterone concentration in patients with PA (94). Of note, ENaC decreased during treatment with mineralocorticoid receptor antagonists and after adrenalectomy, while plasma aldosterone concentration diminished only after surgery treatment, indicating that ENaC reflects mineralocorticoid receptor activity during PA therapy (94).

Ma et al. studied the role of wolframin, a transmembrane protein, which maintains calcium homeostasis by promoting calcium transport from endoplasmic reticulum to cytoplasm, in PA (95). The investigators assessed the proteome and phosphoproteome of tumour tissues from 15 patients with APA and 10 patients with nonfunctioning adrenocortical tumours, applying a 4D label-free quantification approach by high-resolution liquid chromatography-mass spectrometry (95). The results of this study led to the generation of proteome and phosphoproteome signalling network maps of APA and identification of wolframin as a relevant regulatory protein in PA (95).

**Figure 3:**
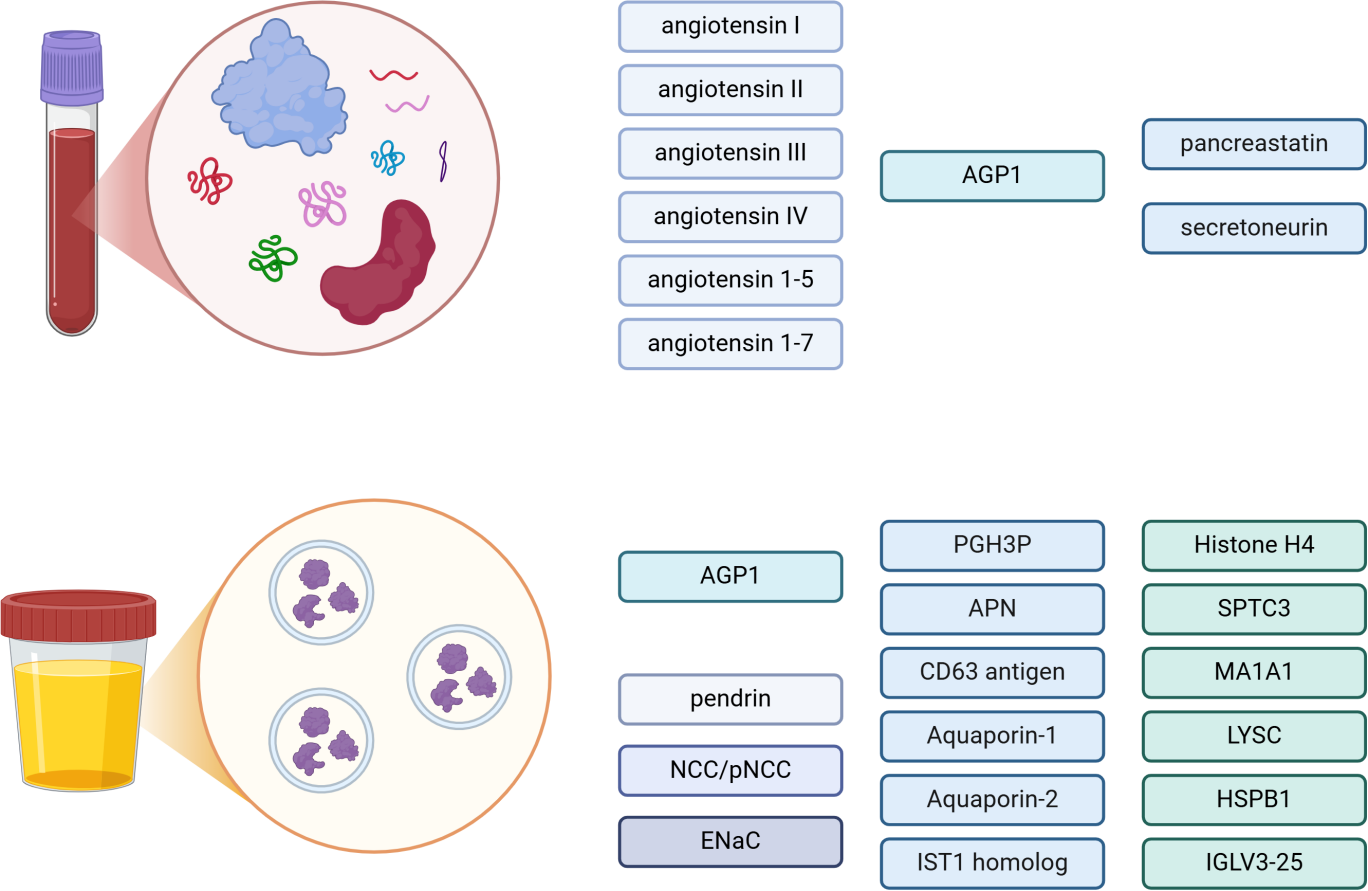
Proteins and peptides in blood and urinary extracellular vesicles (EVs) – potential biomarkers in diagnostics of primary aldosteronism. AGP1, alpha-1-acid-glycoprotein; NCC, Na+Cl- cotransporter; pNCC, phosphorylated Na+Cl- cotransporter; ENaC, γ-epithelial sodium channel; PGH3P, putative glutathione hydrolase 3 proenzyme; APN, aminopeptidase N; SPTC3, serine palmitoyltransferase 3; MA1A1, mannosyl-oligosaccharide 1,2-alpha-mannosidase IA; LYSC, lysozyme C; HSPB1, heat shock protein beta-1; IGLV3-25, immunoglobulin lambda variable 3-25. Created in BioRender. BioRender.com/e86w482

Another proteomic analysis comparing uEVs derived from patients with essential hypertension or PA revealed six proteins (putative glutathione hydrolase 3 proenzyme, aminopeptidase N, CD63 antigen, aquaporin-1, IST1 homolog and aquaporin-2) distinguishing these two groups (88). Interestingly, a reduced abundance of membrane aquaporins involved in water reabsorption mechanisms in PA was observed, which might be explained by chronic water and sodium retention in PA due to the aldosterone excess, when compared with the patients with essential hypertension (88). The statistical analysis also showed usefulness of the following markers: histone H4, serine palmitoyltransferase 3 (SPTC3), mannosyl-oligosaccharide 1,2-alpha-mannosidase IA (MA1A1), lysozyme C (LYSC), heat shock protein beta-1 (HSPB1) and immunoglobulin lambda variable 3-25 (IGLV3-25), in differentiating BPA from APA (88). Proteins and peptides assessed in blood and in urinary EVs in diagnostics of PA are presented in **Figure 3**.

Proteomics research has allowed us to analyze not only a large number of proteins at the given time, but also their properties, abundance and structures. In PA, proteomic studies may help to reliably assess RAAS function, disease subtype, complications and response to the treatment.

## 6 Conclusions

Despite huge advances in the diagnosis and treatment of adrenal disease, PA still lags behind as the most common cause of secondary hypertension with still only a small percentage of patients diagnosed and successfully treated. The complex biochemical assessment of PA may be challenging since it abounds in pitfalls from the screening, confirmatory testing to disease subtyping. The limitations of routine diagnostics of PA necessitates establishing novel diagnostic strategies. Recent development in LC-MS/MS technique and “omics” techniques, including steroidomics and proteomics, offers unique insights into PA mechanism, subtype, molecular background, and even complications. However, these techniques have limitations to be regarded such as limited availability, high cost, often laborious sample preparation and large amount of generated data that must be interpreted cautiously.
